# Micro-offline gains do not reflect offline learning during early motor skill acquisition in humans

**DOI:** 10.1073/pnas.2509233122

**Published:** 2025-10-28

**Authors:** Anwesha Das, Alexandros Karagiorgis, Jörn Diedrichsen, Max-Philipp Stenner, Elena Azañón

**Affiliations:** ^a^Leibniz Institute for Neurobiology, Magdeburg 39118, Germany; ^b^Department of Neurology, Faculty of Medicine, Otto-von-Guericke University, Magdeburg 39120, Germany; ^c^Western Center for Brain and Mind, Western University, London, Ontario N6A 3K7, Canada; ^d^Computer Science, Western University, London, Ontario N6A 3K7, Canada; ^e^Departments of Statistical and Actuarial Sciences, Western University, London, Ontario N6A 3K7, Canada; ^f^Center for Behavioral Brain Sciences, Magdeburg 39106, Germany; ^g^Center for Intervention and Research on adaptive and maladaptive brain Circuits underlying mental health, Jena-Magdeburg-Halle 39106, Germany; ^h^Faculty of Psychology, Otto-von-Guericke University, Magdeburg 39106, Germany

**Keywords:** micro-offline gains, motor sequence learning, offline learning, micro-consolidation, early motor learning

## Abstract

While practicing a new motor skill, resting for a few seconds can improve performance immediately after the rest. This improvement, referred to as “micro-offline gains” (MOGs), has been interpreted as rapid offline learning and is thought to be supported by neural replay of the trained movement sequence during rest. Here, we provide evidence that MOGs reflect only transient performance benefits, partially mediated by motor planning, and not offline learning. In five experiments, participants trained to produce a sequence of finger movements as many times as possible. When participants trained during 10-s practice periods, interleaved with 10-s rest periods, they produced more correct keypresses during training than participants who trained continuously without taking breaks. However, this benefit vanished within seconds after training, when both groups performed under comparable conditions, revealing similar levels of skill acquisition. This challenges the idea that MOGs reflect offline learning, which, if present, should result in sustained performance benefits, compared to training without breaks. Furthermore, we observed persistent MOGs even when participants produced random, nonrepeating sequences, indicating that MOGs do not relate to the offline consolidation of a sequence-specific memory. Rather, sequence-specific learning was only evident online, during practice. Finally, MOGs were diminished when participants could not preplan the first few movements of an upcoming practice period. Our results suggest that MOG are simply an effect of slowing during practice, combined with the possibility of preplanning the initial movements after rest, and therefore do not serve as a reliable metric for offline learning or skill-related consolidation.

## Significance

Recent studies have claimed that motor memory consolidation can occur within seconds of rest interspersed with practice periods, during early skill training. Our findings call for a reconsideration of the idea of micro-offline consolidation and the role of short rest during early motor sequence learning. We show that short breaks lead to transient performance improvements, and not true offline learning. Furthermore, these gains also occur with random sequences, which cannot be learned, and they are partially driven by preplanning. Given the attractiveness of “learning while resting,” these findings demonstrate that micro-offline gains are not a valid indicator of offline consolidation, with important implications for how early skill acquisition is conceptualized.

It has long been acknowledged that breaks between training sessions can enhance learning in cognitive tasks (1–3). However, while there is extensive research on spacing effects on the acquisition of declarative knowledge (4), from vocabulary learning (5) to mathematics (6) (for a review, see ref. 7), the effects of spacing on motor skill acquisition (8–12), particularly on motor sequence learning (11), has received comparatively little attention. Recent findings, however, suggest a beneficial effect of short breaks, in the order of a few seconds, on early motor skill acquisition. Specifically, when humans train to repeat a sequence of finger movements as often and as accurately as possible during repeated 10-s practice periods, alternating with 10-s rest periods, they often execute the first correct sequence in a practice period faster than the last correct sequence in the preceding practice period. Previous studies (13–15) have interpreted these so-called “micro-offline gains (MOGs)” as a behavioral readout of sequence-specific offline learning and claimed that offline learning almost entirely accounts for early skill acquisition, with negligible contribution from online processes during active practice. This assumption has shaped the dominant interpretation of MOGs in the recent literature (16–18).

Widely used in studies (16–24) nowadays, as a measure of rapid offline learning, this counterintuitive idea has also inspired investigations into the neural processes that occur during rest and might drive MOGs. Studies in humans (15, 19, 22) and nonhuman primates (17) have reported that sequence-specific neural activity patterns observed during practice re-emerge during rest. These neural events during rest have been interpreted as replay (15), or reactivation (17, 22), of the trained movement sequence, driving offline learning, and have been linked to MOGs. Crucially, this interpretation rests on the assumption that MOGs reflect sequence-specific offline learning.

However, learning is but one of several factors that jointly influence the dynamics of motor performance over time (10, 25). An intuitive effect of taking a break is recovery from fatigue (26), or reactive inhibition (27, 28)—a transient, task-induced suppression of motor output that accumulates during repetitive motor tasks and reduces performance independently of learning. Once the task stops, suppression dissipates, and performance recovers. For rapid, repetitive finger movements, fatigue starts to slow down motor performance after as little practice time as 10-s (29, 30). Thus, fatigue as well as reactive inhibition can mask the true skill level acquired by the end of a practice period (25, 29, 31, 32). Therefore, MOGs may simply reflect the dissipation of fatigue (29, 30) following a break, unmasking the skill level already reached at the end of the preceding practice period. In fact, recent literature has already provided evidence that dissipation of reactive inhibition contributes to MOGs (32).

Besides recovery from fatigue and reactive inhibition, rest provides an opportunity for planning. When people prepare to restart a motor sequence following rest, they typically preplan several movements in advance (33–35). Preplanning enhances initial motor performance. Thus, when training with breaks, the dynamics of motor performance over time reflect the net effect of learning, recovery of reactive inhibition, and planning.

Despite these well-established effects, previous investigations into MOGs have largely ignored the influence of fatigue and preplanning. Instead, they have hypothesized MOGs to be a form of microconsolidation, based on processes (15, 17, 22) that operate entirely during the rest. This is surprising because MOGs—defined as the performance improvement from the last correct sequence before rest to the first correct sequence after rest—are computed from performance data generated entirely during practice, not rest. Moreover, there is no compelling evidence for the proposed processes of neural replay or reactivation (15, 17, 22) to have any causal effect on MOGs, under controlled conditions. Given the absence of a baseline for performance that is uncontaminated by fatigue or reactive inhibition, and matched for preplanning, the assumption that any postrest performance improvement is due to microconsolidation, is problematic.

If MOGs reflect offline learning, i.e., learning that occurs only during breaks, then training with breaks should result in a higher skill level than training without breaks. Furthermore, if offline learning is sequence-specific, as suggested by the idea of sequence-specific replay (15) or reactivation (17, 22), offline learning should not transfer to novel sequences which were never trained before.

Here, we tested these critical predictions in six behavioral experiments. We provide evidence that MOGs are neither sequence-specific, nor associated with enhanced skill acquisition. Within a practice period, true online learning is masked by fatigue (29–31) and reactive inhibition (32), leading to slowing of performance. When given a break, there is a chance for recovery as well as the possibility to preplan the first few keypresses, upon task reinitiation right after the break. Therefore, the postbreak performance gains, i.e., MOGs, likely reflect true online learning after recovery from fatigue and reactive inhibition, combined with preplanning. This has crucial implications for interpreting physiological signals that occur during rest.

## Results

### Experiments 1 and 2 (and S1): MOGs Do Not Reflect Offline Learning.

We tested whether people reach higher skill levels when training with breaks, compared to training without breaks, in an in-lab study (Experiment 1, N = 85 across two groups), and confirmed results in a larger cohort via an online study (Experiment 2, N = 358 across two groups). Since MOGs are predominantly observed during early learning, defined in previous studies as the first trial to the 11th trial of training (13–15), our study specifically targeted this early learning phase.

**Fig. 1.**
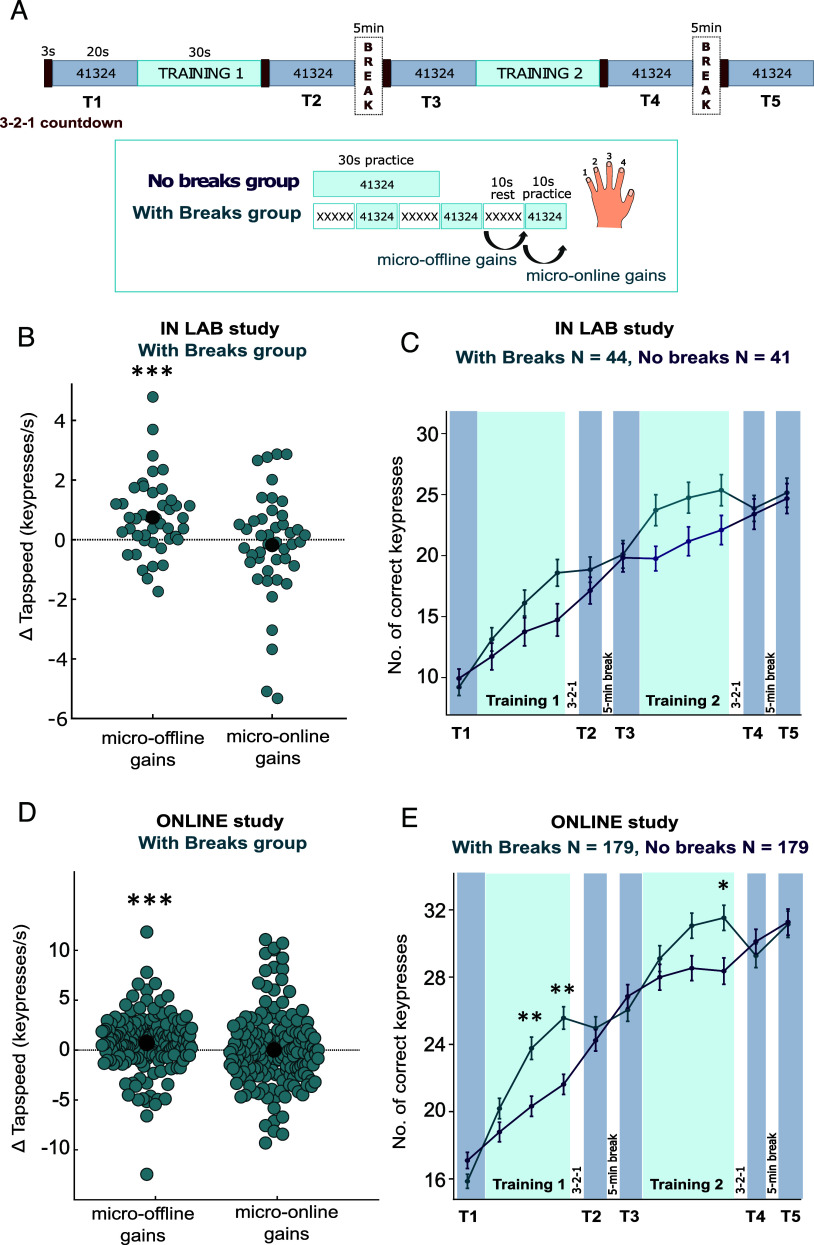
MOGs do not reflect offline learning: Experiment 1 (in-lab study) and Experiment 2 (online study). (*A*) Experimental design. Both groups trained to produce the sequence 4-1-3-2-4 as accurately and as often as possible throughout fixed-duration practice periods, distributed across two training blocks (Training 1 and Training 2). One group of participants (“No Breaks”) trained to produce the sequence via continuous practice periods of 30 s each, whereas the other group (“With Breaks”) trained for the same total amount of time broken into three 10-s practice periods, interleaved with 10-s rest periods. In both groups, we assessed skill level at five time points during the experiment, via test sessions of 20-s each (T1–T5). The task was identical for practice periods and test sessions. Panels *B* and *C* show data from the in-lab study (Experiment 1), while (*D* and *E*) show data from the online study (Experiment 2). In the group with breaks, we computed micro-online and MOG as defined in previous studies (13–15). Micro-online gains are the difference in tapping speed between the last and first correct sequence within a practice period. MOG are the difference in tapping speed between the last correct sequence of one practice period and the first correct sequence of the next practice period. Summing these changes across 6 trials of two training blocks, yields the total online gains and total offline gains per participant, respectively. We obtained similar results when computing micro-online gains and MOG separately for each training block (*SI Appendix*, Fig. S1 *F* and *L*). To allow for between-group comparison (panels *C* and *E*), we binned performance during the 30-s practice periods of the group without breaks into 10-s bins. For illustration purposes and baseline (T1 and T2) for each training, the 20-s test sessions were split into 10-s bins and averaged. Error bars represent SEM. Asterisks indicate levels of statistical significance: *** p <0.001, ** p<0.01, * p<0.05. See also *SI Appendix*, Fig. S1 for additional analyses, and *SI Appendix*, Fig. S2 for an additional experiment.

As in previous MOGs studies (13–15), right-handed participants trained to produce a sequence of five finger movements as often and as accurately as possible throughout fixed-duration practice periods, using their left hand (“4-1-3-2-4,” where “1” represents the little finger and “4” the index finger). We divided participants into two groups with different training schedules. One group trained with interleaved 10-s rest periods, while the other group trained without interleaved rest (Fig. 1*A*). We evaluated skill level at five time points during the experiment (T1–T5) via 20-s test sessions. Note that test sessions were essentially continuous 20-s practice periods in which participants performed the same 4-1-3-2-4 sequence as frequently and accurately as possible, just as during training. In addition, to ensure identical test conditions across groups, each of the five test sessions (T1–T5) was preceded by a 3-s countdown on the screen.

The procedure for both groups began with a baseline test (T1) lasting 20-s, followed by the first block of training (Training 1). In the group with breaks, training consisted of three 10-s practice periods, each preceded by a 10-s rest period. In contrast, the no-break group transitioned directly from the baseline test to a continuous 30-s practice period without rest. In both groups, training concluded with a 3-s countdown displayed on the screen, signaling participants to stop their movements and prepare for the upcoming 20-s test session (T2). This ensured that both groups started the test session under comparable conditions. After T2, participants took a 5-min break before completing a 20-s retention test (T3). To assess group differences arising at a slightly later stage of skill acquisition, participants completed a second training block, designed similarly to the first. For the group with breaks, the second training block consisted of three 10-s practice periods, each preceded by a 10-s rest period. For the group without breaks, T3 transitioned directly to a continuous 30-s practice period without rest. In both groups, the second training concluded with a 3-s countdown, followed by a 20-s test session (T4), a 5-min break, and a final 20-s retention test (T5).

We first confirmed that the group who trained with breaks exhibited MOGs, defined identically to previous studies, i.e., greater tapping speed for the first correct sequence in a 10-s practice period than for the last correct sequence of the preceding practice period. We observed significant MOGs, during training blocks, both in the in-lab study (t(43) = 3.92, *P* < 0.001, d = 0.59, BF_10_ = 84.73; Fig. 1*B*), and in the online study (t(178) = 3.53, *P* < 0.001, d = 0.26, BF_10_ = 30.99; Fig. 1*D*; one-sample *t* tests against zero; See *SI Appendix*, Fig. S1 *F* and *L* and Table S2 for results on separate training blocks). Additionally, we calculated micro-online gains, defined in prior studies as the difference between the first and last correct sequence of a 10-s practice period. These were not statistically significant (all *P* ≧ 0.165; *SI Appendix*, Table S2).

To compare training performance between groups, we binned the continuous 30-s practice periods in the group without breaks into 10-s bins (Fig. 1 *C* and *E*). Training performance—measured as the number of correct keypresses during training—improved more in the group with breaks than in the group without breaks. For Training 1, a 2 × 4 ANOVA (group: with breaks vs. no breaks; time points: T1 baseline (averaged over two 10-s bins) and three training bins) revealed a main effect of time point (in-lab: F(2.4,199.8) = 78.54, *P* < 0.001, η^2^_partial_ = 0.49; online: F(2.9,1036.9) = 189.82, *P* < 0.001, η^2^_partial_ = 0.35). A significant group by time point interaction was also observed (in-lab: F(2.4,199.8) = 7.60, *P* < 0.001, η^2^_partial_ = 0.08; online: F(2.9,1036.9) = 27.19, *P* < 0.001, η^2^_partial_ = 0.07). This indicates that performance improved during training in both groups, but to a greater extent, in the group with breaks. Performance during the second training block showed a similar pattern (*SI Appendix*, Table S1). For *t* test comparisons across groups at each time point, refer to *SI Appendix*, Table S1.

Despite the performance difference during training and the presence of MOGs in the group with breaks, both groups ultimately reached a similar skill level: The number of correct keypresses in all 20-s test sessions was comparable between groups (Fig. 1*C*). A 2 × 5 ANOVA (group: with breaks vs. no breaks; test sessions: T1–T5) showed significant improvements across test sessions in both the in-lab study (F(2.9, 238.3) = 250.08, *P* < 0.001, η^2^_partial_ = 0.75) and the online study (F(3.2, 1141.4) = 591.54, *P* < 0.001, η^2^_partial_ = 0.62), but no significant main effect of group (in-lab: F(1,83) = 0.09, *P* = 0.761, η^2^_partial_ < 0.01; online study: F(1, 356) = 0.30, *P* = 0.584, η^2^_partial_ < 0.01). A small, but significant group × test session interaction was observed in the online study (F(3.21, 1141.36) = 2.92, *P* = 0.030, η^2^_partial_ < 0.01), but not the in-lab study (F(2.9, 238.3) = 0.78, *P* = 0.504, η^2^_partial_ < 0.01). Critically, independent-samples *t* tests revealed no significant performance differences between groups for any test session in either study (all p_corr_ > 0.2, all BF_10_ ≤ 0.97; independent-samples *t* tests; detailed results in *SI Appendix*, Table S1).

To corroborate that the absence of group differences was not due to differences in the design of our experiments and previous MOGs studies, specifically the inclusion of 20-s test sessions before and during learning, we performed a replication of the original paradigm by Bönstrup et al. (13) (*SI Appendix*, Fig. S2), testing the first seven trials. One group trained in 10-s practice periods, interleaved with 10-s rest periods (n = 31), while the other group trained continuously for 70 s (n = 31). Again, we observed no group differences in performance (i.e., number of correct keypresses) following a 3 min rest after training, despite the presence of MOGs in the group with breaks (*SI Appendix*, Table S3). In addition, across Experiments 1, 2, and S1, we found no group differences in number of correct sequences, tapping speed, or percentage of correct keypresses during the test phases (*SI Appendix*, Figs. S1 and S2 and Tables S2, S3 and Accuracy metrics). This consistency, along with the fixed 10-s trial duration and explicit instructions to maximize correct sequences, limits the possibility of hidden speed–accuracy trade-offs. Moreover, no performance difference was found between participants tested with or without performance-related monetary incentive (*SI Appendix*). This suggests that the observed absence of group differences after training, are not in any way related to the fact that participants in Experiments 1, 2, and S1 received bonus payment based on their performance.

In summary, taking short breaks resulted in immediate performance benefits during training (Fig. 1 *B*–*E*), as reflected in a larger number of correct keypresses produced at the end of training. However, this benefit disappeared within seconds after training, with comparable skill levels across groups both in the test sessions completed after the 3-s countdown (T2 and T4), and in the retention tests conducted after 5-min breaks (T3 and T5; Fig. 1 *C* and *D*). Importantly, in spite of MOGs exhibited by the group who trained with breaks, their skill level was comparable to the group that did not have the chance to exhibit MOGs, due to training without breaks.

### Experiment 3: MOGs Do Not Reflect Sequence-Specific Learning or Consolidation.

**Fig. 2.**
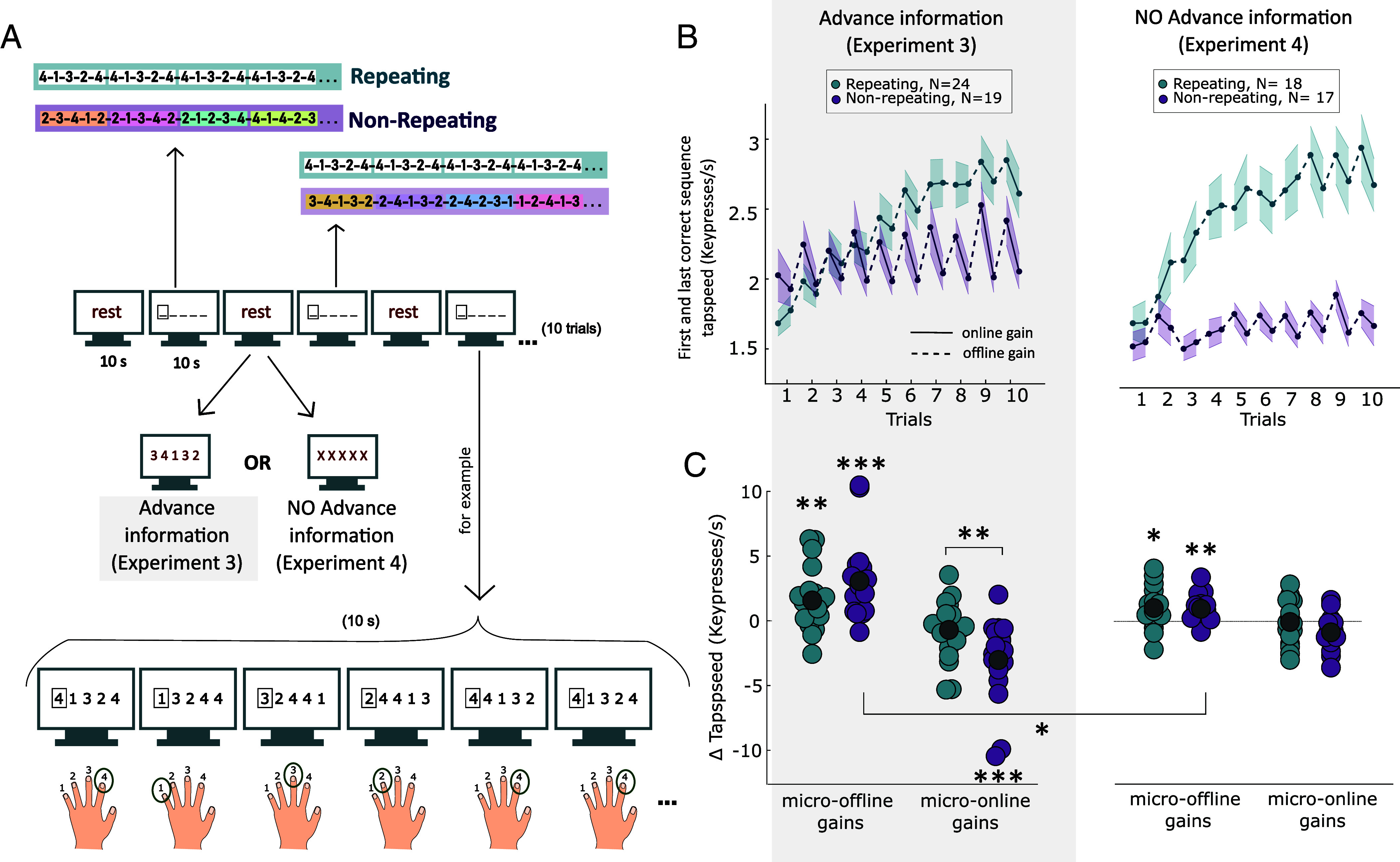
MOGs do not reflect sequence-specific learning: Experiment 3 (advance information) and Experiment 4 (no advance information). (*A*) Experimental design: In 10-s practice periods, participants saw a string of five numbers, cueing a sequence of five keypresses. A rectangle surrounded the leftmost number, cueing the next movement, similar to a Discrete Sequence Production (DSP) task. Each keypress shifted the numbers leftward, revealing a new number in the fifth position. The Repeating group saw the same five-element sequence repeatedly (4-1-3-2-4), which allowed for sequence-specific learning, while the Nonrepeating group performed novel, nonrepeating five-element sequences, thereby preventing sequence-specific learning. The colored or white boxes shown in panel A to separate consecutive five-element sequences are for illustration purposes and were not shown to participants. A 10-s rest preceded every practice, displaying either the next five-element sequence (advance information, Experiment 3), or “XXXXX” (no advance information, Experiment 4). (*B*) Tapping speed across the ten trials (10-s practice periods) of each experiment. For each trial, we show the speed for the first and last correct sequence. Dotted lines indicate MOG, whereas solid lines indicate micro-online gains. (*C*) In both experiments, Repeating and Nonrepeating groups showed MOGs of comparable size. For the Nonrepeating group, MOGs were significantly larger in Experiment 3 advance information), compared to Experiment 4, where advance planning of upcoming sequence was prevented. All error bars represent the SEM. Asterisks indicate levels of statistical significance: *** p <0.001, ** p<0.01, * p<0.05.

We next tested whether MOGs reflect sequence-specific learning, as predicted by the idea of replay, or reactivation, of the trained sequence during rest (15, 17). To test this, Experiment 3 compared MOGs in participants who practiced a single, repeating sequence 4-1-3-2-4 (Repeating group, N = 24 participants) with participants who produced five-element sequences that never repeated (Nonrepeating group; N = 19 participants; Fig. 2*A*). Both groups alternated between 10-s practice periods and 10-s rest periods for 10 trials. To ensure equivalent advance information about the upcoming sequence, the first sequence of the upcoming practice period was displayed on the screen throughout the preceding rest period for both groups.

As expected, we observed sequence-specific learning in the Repeating group, but not in the Nonrepeating group, as shown by a significant group × trial (10 trials) interaction effect on the number of correct keypresses (F(3.6, 146.9) = 13.75, *P* < 0.001, η^2^_partial_ = 0.25; *SI Appendix*, Fig. S4). Importantly, we found significant MOGs (Fig. 2 *C*, *Left*) in both groups (Repeating group: t(23) = 3.53, *P* = 0.002, d = 0.72, BF_10_ = 21.22, Nonrepeating group: t(18) = 4.52, *P* < 0.001, d = 1.04, BF_10_ = 117.14; one-sample *t* tests against zero). Furthermore, the magnitude of MOGs was similar in the two groups (t(41) = −1.90, *P* = 0.968, d = −0.58; independent-samples *t* test, one-sided, i.e., H_1_: Repeating Group > Nonrepeating group), and Bayesian analysis provided moderate evidence in favour of the null hypothesis (BF_10_ = 0.12). Instead, the difference between the Repeating and Nonrepeating groups arose during ongoing practice, i.e., in “micro-online gains” (Fig. 2 *C*, *Left*, t(41) = 3.00, *P* = 0.005, d = 0.92, BF_10_ = 9.08; independent-samples *t* test two-sided).

A closer examination of this effect is presented in Fig. 2 *B*, *Left*, which displays the speed of the first and last correct sequences used to calculate the MOGs. MOGs are represented by dotted lines, and micro-online gains are shown by shaded lines, for each trial. In the Nonrepeating group, tapping speed declined during the course of each 10-s practice period. This slowing during practice was followed by an initial postrest performance boost, giving rise to the observed MOGs. In the Repeating group, the decline in tapping speed during practice was less pronounced, likely because it was balanced by sequence-specific learning during practice.

In summary, the results of Experiment 3 reveal that MOGs do not reflect sequence-specific learning, because MOGs are equally present when training to produce novel, nonrepeating sequences. Instead, sequence-specific learning occurs during practice, not rest, as indicated by group differences in the dynamics of tapping speed across practice. While the Nonrepeating group revealed pronounced motor slowing during practice, the Repeating group could balance motor slowing with online learning. This contrasts with the idea that, when training with breaks, early learning occurs exclusively during rest, as has been explicitly proposed in previous studies (13–18).

### Experiments 4 and 5: MOGs Partially Reflect Motor Preplanning.

Breaks provide an opportunity for planning (33, 34). In Experiment 3, both groups were cued with the upcoming sequence during the preceding rest period, allowing them to preplan the first few elements of the sequence before initiating the first movement in each practice period. For the Repeating group, the cue served primarily as a reminder, as they could already anticipate the fixed sequence. Given that preplanning accelerates the first three to four keypresses, after which execution slows as remaining elements are planned online (33–35), this likely contributed to the observed MOGs.

Experiment 4 tested the role of preplanning in MOGs by removing any advance cueing of the upcoming sequence, thus reducing the opportunity for extended preplanning during the rest period (N = 18 for the Repeating group and N = 17 for the Nonrepeating group). Removing this opportunity was expected to reduce the MOGs for the Nonrepeating group, which could not rely on the advance information to be able to plan ahead (as in Experiment 3). However, even for this group some MOGs may remain, as participants can still preplan the initial elements of a sequence during the brief interval between the presentation of the sequence cues on the screen (which signals the start of the practice period) and movement initiation (i.e., the reaction time) (34).

As in Experiment 3, performance improved more in the Repeating group than the Nonrepeating group, as shown by a significant group × trial (10 trials) interaction on the number of correct keypresses (F(4.7, 155.3) = 12.06, *P* < 0.001, η^2^_partial_ = 0.27, *SI Appendix*, Fig. S4). Both groups again showed significant MOGs (Repeating: t(17) = 2.85, *P* = 0.011, d = 0.67, BF_10_ = 4.84; Nonrepeating: t(16) = 4.02, *P* < 0.001, d = 0.98, BF_10_ = 38.26; one-sample *t* tests against zero), and the magnitude of MOGs did not differ between them (t(33) = 0.15, *P* = 0.885, d = 0.05, BF_10_ = 0.33; independent-samples *t* test), replicating the finding that MOGs also occur for nonrepeating sequences.

Critically, MOGs in the Nonrepeating group were reduced relative to the same group in Experiment 3, where preplanning during rest was possible (Fig. 2*C*, t(22.4) = 2 .91, *P* = 0.008, d = 0.95, BF_10_ = 11.20, Welch’s *t* test, Bayesian Welch’s *t* test, to account for unequal variances across groups). This suggests that the measure of MOGs is sensitive to explicit cueing of upcoming movements during rest and may partially reflect motor preplanning.

Although Experiment 4 eliminated the possibility of preplanning in the rest-periods, preplanning during the reaction time remained possible (34). In fact, in the Nonrepeating group in Experiment 4, initiation time was significantly delayed (884 ± 188 ms, mean ± SD), compared to the Nonrepeating group in Experiment 3 (585 ± 172 ms; t(34) = 5.00, *P* < 0.001, d = 1.67, BF_10_ = 970.77; independent-samples *t* test), based on practice periods that began with a fully correct sequence (90.49% of practice periods). This reaction time cost likely reflects planning at the onset of the practice period, potentially contributing to the persistent—though reduced—MOGs. Nonetheless, one should note that such cross-experiment between-subject comparison limits interpretation.

**Fig. 3.**
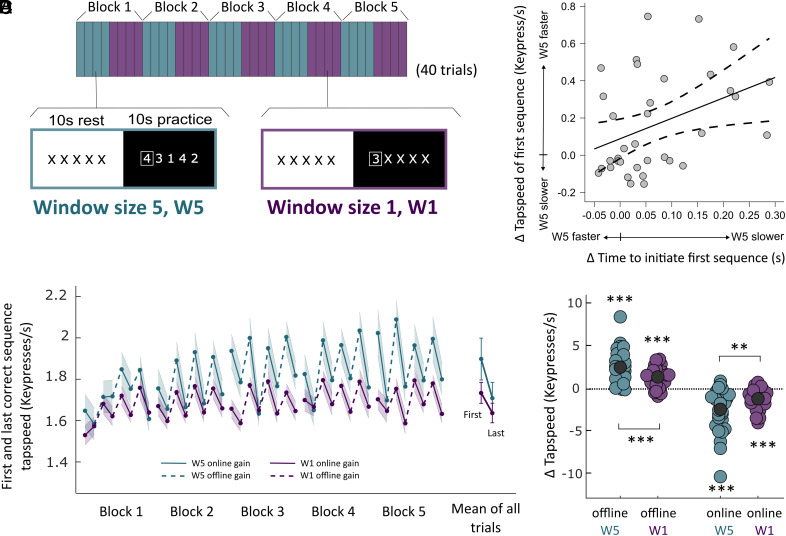
MOGs partially reflect motor preplanning: Experiment 5. (*A*) Experimental design. In this within-subject experiment, two conditions [Window size 5 (W5) and Window size 1 (W1)] alternated every four trials, where a trial was defined as a 10-s practice period, together with the preceding 10-s rest period. During practice, the screen displayed a string of five numbers (W5 condition), or a string consisting of a single number followed by four X’s (W1 condition). A rectangle highlighted the leftmost number in the string (i.e., the single number in the W1 condition) as the cue for the next movement, similar to a DSP task. Across practice, participants had to perform nonrepeating five-element sequences of movements, similar to the Nonrepeating group in Experiments 3 and 4. Because the string consisted of five numbers shown simultaneously in the W5 condition, participants could preplan up to five finger movements. This preplanning could only occur at the start of the 10-s practice period, as each practice period was preceded by a 10-s rest period showing “XXXXX.” In the W1 condition, on the other hand, only the single next number was shown (and updated with every new keypress), followed by four X’s. This prevented planning of more than a single finger movement at a time. (*B*) Tapping speed of the first and last correct sequences for both conditions in each block. Dotted lines indicate MOG, whereas solid lines indicate micro-online gains. Increasing the window size (from the W1 condition to the W5 condition) resulted in performance benefits that were stronger for the first correct sequence than for the last correct sequence. (*C*) Difference in tapping speed between the two conditions (W5 minus W1) plotted against the difference in time to initiate the first sequence after a rest (W5 minus W1), for each participant. Consistent with preplanning, participants traded-off the speed of initiating the first movement in a practice period, and the tapping speed of the first correct sequence. Participants with slower movement initiation in the W5 condition, compared to the W1 condition, were faster in executing the first correct sequence in the W5 condition, compared to the W1 condition, and vice versa. (*D*) We found significant MOGs in both conditions, however, MOGs were significantly smaller in the W1 condition. This confirms that preplanning contributes to MOGs. Asterisks indicate levels of statistical significance: *** p <0.001, ** p<0.01, * p<0.05.

To directly assess the contribution of preplanning to MOGs, we conducted a final, within-subject experiment that systematically manipulated the planning horizon (Experiment 5, N = 35, Fig. 3*A*). Participants produced nonrepeating sequences in two conditions. In one condition, we cued only one key (number) at a time, so that participants could not preplan beyond the single next movement (“Window size 1”, W1). In the other condition, we simultaneously cued the next five keys (numbers), so that participants could preplan up to five movements at a time (“Window size 5”, W5). Participants received no advance information during rest, so that any preplanning was restricted to the reaction time interval, i.e., the time interval between the start of the practice period and the first keypress.

Consistent with preplanning, initiation of the first movement after a break were significantly slower in W5 trials (796 ± 126 ms, mean ± SD) compared to W1 trials (728 ± 122 ms; t(34) = 4.34, *P* < 0.001, d = 0.73, BF_10_ = 212.87, considering only those practice periods that started with a fully correct sequence (87.21% and 83% of W5 and W1 trials respectively). This delay allowed participants to be faster in executing the first correct sequence (t(34) = 3.66, p_corr_ < 0.001, d = 0.62, BF_10_ = 36.87, comparing W5 and W1), giving rise to a significant interaction between condition (W5 vs. W1) and position (first vs. last correct sequence; F(1,34) = 12.11, *P* = 0.001, η^2^_partial_ = 0.26; Fig. 3*B*). This was also supported by a significant correlation between costs in initiation time and benefit in the tapping speed of the first sequence (r = 0.39, *P* = 0.022; Fig. 3*C*), in the two conditions.

As a consequence of the larger window size, MOGs were significantly larger in the W5 condition than in the W1 condition (t(34) = 3.69, *P* < 0.001, d = 0.62, BF_10_ = 39.25; Fig. 3*D*). However, preventing preplanning did not completely abolish the slowing during the 10-s practice period. Significant MOGs were still present in both conditions when tested against zero (t(34) = 8.24, *P* < 0.001, d = 1.39, BF_10_ = 8.876 × 10^6^, W5 condition; t(34) = 7.85, *P* < 0.001, d = 1.33, BF_10_ = 3.16 × 10^6^, W1 condition). This indicates that although preplanning contributes to MOGs, other processes such as release from reactive inhibition (32) or fatigue (29–31) likely also play a role.

## Discussion

Our results challenge the prevailing view that MOG reflect offline learning or consolidation (13–16, 19, 20). We found that training with breaks gives rise to MOG, but conveys no lasting learning benefit over training without breaks, casting doubt on the idea that MOG result from additional (offline) learning during breaks (13–15). Furthermore, we found equivalent MOG during the production of random, nonrepeating sequences. This challenges the idea that MOG reflect sequence-specific learning, and casts doubt on previous proposals that MOG are driven by neural events during rest interpreted as replay or reactivation of a trained sequence (15, 17).

Instead, our data indicate that the most parsimonious explanation for MOGs is that they reflect transient dynamics in skill expression around the time of a break, rather than offline skill acquisition. Studies that interpret MOGs as a behavioral marker of offline learning assume that performance remains stable throughout the practice period. However, at any given skill level, performance varies over time, owing to systematic task-related factors, such as fatigue, reactive inhibition or planning, the latter of which was directly demonstrated in Experiment 5. When performance declines across the practice period, the definition of MOGs underestimates online learning while inflating the apparent benefits of rest. Indeed, across Experiments 3, 4, and 5, the groups performing nonrepeating sequences exhibited slowing of motor performance during practice (Figs. 2*B* and 3*B*). Such slowing could arise due to a switch from fast preplanned movements to slower online planning (33), or due to fatigue (29, 30, 36) or reactive inhibition (32). In line with our results, motor slowing has been observed within the first 10-s of practice before (29). When viewed through the lens of online- and offline-gains, motor slowing leads to negative “micro-online gains,” as observed for the Nonrepeating groups in our experiments. There is no reason to assume that the factors that drive such slowing during practice, should not also be present for the Repeating group. Therefore, it is likely that the “zero micro-online gains” observed when people train to produce a single, repeating sequence (13, 14), reflect a net effect of slowing plus concurrent, sequence-specific online learning. As a result, micro-online gains observed for the Repeating group in Experiment 3 were close to zero. Similarly, previous studies have found no net change in performance during 10-s practice periods (13, 15, 19), or a net slowing of performance (19, 20, 22, 37) (as in our conceptual replication of the Bönstrup et al. (13) paradigm, see *SI Appendix*, Fig. S3).

In the presence of factors that deteriorate performance within a practice period, the standard definition of MOGs (i.e., the improvement in performance, from the last correct sequence before rest to the first correct sequence after rest) cannot differentiate offline learning from online learning whose effect on performance becomes evident only after a break. Our findings indicate that MOGs are primarily driven by processes occurring during practice, rather than rest. Sequence-specific learning, as measured by the difference between Repeating and Nonrepeating groups, only occurred during practice. However, after rest, when preplanning becomes possible again and fatigue (29, 30, 36) as well as reactive inhibition (32, 38) have dissipated, these latent learning effects manifest as a temporary performance boost. Thus, sequence-specific learning occurred during practice but became apparent only after rest, giving rise to MOGs. We observed higher MOGs for the Nonrepeating group of Experiment 3 compared to Experiment 4, hinting toward the role of planning during rest periods. However, baseline performance (*SI Appendix*, Fig. S4) differed between the two Nonrepeating groups, perhaps owing to sampling bias.

In summary, while breaks induce MOGs, they do not provide evidence of offline learning. Recognizing the limitation of this measure is therefore crucial for interpreting performance changes in motor learning tasks.

This is especially important given the growing body of research linking MOGs, interpreted as a measure of wakeful consolidation, to neural events during rest (15, 17, 19, 22). For instance, Buch et al. (15) found that MOGs correlated with events in magnetoencephalography (MEG) data during rest, which they interpreted as rapid replay of the trained sequence (see also refs. 17 and 22). If neural replay of the trained sequence were the primary driver of MOGs, one would expect MOGs would at least be smaller when the sequences never repeat during training. However, our findings from Experiments 3 and 4 revealed that MOGs were of similar magnitude, even for novel sequences that never repeated. While our findings do not rule out the possibility of neural replay during rest, they demonstrate that sequence-specific replay is not necessary for MOGs to occur. This highlights the lack of specificity of MOGs as a measure, which is likely a consequence of a general slowing during motor execution, rather than constituting a behavioral index of replay-mediated microconsolidation, as extensively discussed in the literature (13–17, 19, 22, 39)–and should therefore be considered with caution.

Two recent studies by Gupta et al. (32, 38) indicate that reactive inhibition occurs even within 10-s of practice, and that the dissipation of reactive inhibition contributes to postrest performance improvements. This aligns with our conclusion that MOGs are not a specific readout of microconsolidation. Our results extend this view by several critical findings. “Massed” training in Gupta et al.’s studies still included breaks, complicating the interpretation of differences to “spaced” training, while our Experiments 1, 2, and S1 provide an unambiguous test of spacing effects during early motor sequence learning. Furthermore, results from our Experiments 3 and 4 show that MOGs exist even for random sequences. Finally, in Experiment 5, we provide evidence that MOGs are at least partly driven by preplanning. Importantly, Gupta et al. (38) excluded the first completed sequence of each practice period as a “warm-up sequence.” Given that this sequence, together with the last correct sequence in the preceding practice period, defines MOGs, Gupta et al.’s results, although critical for identifying the role of reactive inhibition, do not put MOGs directly to the test.

In functional MRI (fMRI), Blood-Oxygenation-Level-Dependent signals in the hippocampus and precuneus during rest, have been shown to correlate with MOGs (19). Additionally, learning-related patterns in fMRI due to training persist and extend into short interpractice rests (20). While these findings have been interpreted as indicative of a memory reactivation of the sequence trained in the past, they may alternatively reflect planning of future sequence production. Kornysheva et al. (40) identified in MEG, a neural signature of motor sequence planning that involved medial temporal lobe, and that linked practice-related information with the preceding planning stage, broadly consistent with this fMRI findings. They found that several finger movements in an upcoming sequence could be decoded in parallel during preparation. This parallel planning signature has been source-localized to the parahippocampus and cerebellum, and predicted motor sequence performance (35, 40).

Rest interspersed with training could thus provide an opportunity to plan the first elements of the upcoming sequence before restarting training. In that regard, the disruptive impact of theta burst stimulation of the dorsolateral prefrontal cortex (DLPFC) on the extent of MOGs (41) may potentially be attributed to the DLPFC’s critical role in sequential event planning (42, 43). Building on this, in Experiment 5 we observed significantly less MOGs when the preceding rest period, as well as the time before first movement initiation, could not be used for planning several upcoming finger movements, compared to conditions allowing for preplanning. These findings strongly suggest that preplanning contributes to the transient boost in performance after rest, which are reflected as MOGs.

Finally, while spaced training is widely recognized to enhance long-term learning in various domains, including declarative knowledge (4), its effects on motor sequence learning remain insufficiently characterized (8–12). In our study, spacing improved performance during training, but this improvement vanished at subsequent test sessions, within seconds after training. These test sessions allowed for an unbiased comparison of skill levels, and revealed that people acquire similar skill levels whether, or not, they train with breaks. Our findings, therefore, do not support a spacing effect in early motor sequence learning on a short time scale in the order of seconds, at least during the first few trials. Instead, they emphasize an important distinction in motor skill learning, as described by Kantak et al. (25), between motor performance, i.e., observable changes during training, such as MOGs, and motor learning, defined as the longer-term retention and stability of skills developed through training (25, 44, 45).

In conclusion, we propose that the improved performance observed after a short break is short-lived and does not reflect microconsolidation during breaks. The cause of the postrest performance improvements is likely multifactorial, with the ability to preplan movements, playing an important role. Other contributing factors could include the dissipation of reactive inhibition or fatigue during rest. Moreover, attentional recovery facilitated by context switching (46), in conjunction with preplanning, may explain why a pause of only 3 s is sufficient to unveil true performance levels. Our findings highlight the need for researchers to exercise caution when interpreting MOGs. As our work demonstrates, MOGs is not a valid indicator of micro-offline learning and may not be suitable as a proxy for linking behavioral data to neurophysiological processes, particularly in the context of wakeful consolidation during rest.

## Methods

Across the five experiments, a total of 631 participants (270 females) took part in this study. 89 (31 females, mean age = 25.9 y, SD = 3.2 y) took part in Experiment 1 and 413 (175 females, mean age = 27.9 y, SD = 7.7 y) took part in Experiment 2, 49 (29 females, mean age = 39.9 y, SD = 15.8 y) in Experiment 3, 37 (20 females, mean age = 39 y, SD = 13.5 y) in Experiment 4 and 43 (15 females, mean age = 27.3 y, SD = 3.2 y) took part in Experiment 5. For Experiment 1, we calculated the sample size sufficient to detect a difference in the number of correct keypresses between two groups of participants (with and without breaks) that is at least medium-sized (Cohen’s d ≥ 0.6) with at least 80% power [independent-samples *t* test; the pooled SD was estimated from our Experiment S1, tested previously (n = 62 participants; *SI Appendix*, Fig. S2)]. In Experiment 2, our goal was to achieve ≥80% power to observe an effect of group on number of correct keypresses of d ≥ 0.3. Experiments 3 and 4 were crowdsourcing studies during a science communication event at the Leibniz Institute for Neurobiology, Germany (the Magdeburg Long Night of Science), during which we collected data from as many volunteers during the 6 h of the event as possible (total of 86 adult datasets collected). Cohort size for Experiment 5 was determined by our goal to observe any effect of preplanning on MOG of size d ≥ 0.5 with ≥80% power.

Participants in Experiments 1 and 5 were recruited based on the following exclusion criteria. They had to be right-handed, between 18 and 40 y of age, could not be professional typists or skilled musicians (i.e., recruited participants did not play any musical instrument requiring skilled finger movements for consecutive 4 y at any point in their life), did not have any prior or existing neurological or psychiatric conditions, and were naïve to the task. Participants for Experiment 2 (online, crowdsourced) were recruited via the following criteria: they had to be right-handed, between 18 to 40 y of age, and without prior neurological or psychiatric condition. There were no criteria applied for participation in Experiments 3 and 4, but we performed analysis only on the data of participants who were right-handed and 18 y of age and above. Participants for the in-lab studies were recruited via local participant databases (Sona systems, https://magdeburg.sona-systems.com/) whose members are mostly students and staff of Otto-von-Guericke University in Magdeburg, whereas participants for the online study (Experiment 2) were recruited via the Prolific database (www.prolific.com). Participants for Experiments 3 and 4 were volunteers during a science communication event, as described above.

Handedness was determined by the Edinburgh Handedness Inventory assessment (Oldfield et al. (47)) in all experiments except Experiment 2, in which right-handedness was self-reported via Prolific. The study was approved by the ethics committee of the Otto-von-Guericke University Magdeburg, Germany, and conducted in accordance with the Declaration of Helsinki. All participants, except for visitors of the Long Night of Science taking part in Experiments 3 & 4, received financial reimbursement for their time of participation, and were additionally rewarded with bonus money based on their performance in the task. All participants provided written informed consent prior to participation and all data were collected and processed in accordance with the General Data Protection Regulation (GDPR).

### Apparatus.

All experiments except the online crowdsourced study were conducted in a behavioral laboratory (a room with four computers on desks, and chairs arranged in the form of cubicles, for participants to sit and perform the task in privacy without distraction) using LCD displays, PC keyboards, and headphones. Stimuli for Experiments 1 and 3-5 were presented using MATLAB (R2021b, The MathWorks, Inc., Natick, Massachusetts, United States) and Psychtoolbox (48), whereas Experiment 2 (the online study) was programmed in PscyhoPy (49) and conducted on Pavlovia (www.pavlovia.org), with participants recruited via Prolific (www.prolific.com). In the laboratory, we tested either single participants, or up to four participants at a time simultaneously.

### General Task Design.

Participants were asked to sit in front of an LCD monitor (60 Hz) and place the little finger, ring finger, middle finger, and index finger of their left hand on four keys of the keyboard. Throughout all experiments except Experiment 2, we used the keys F, T, Z, and J (on a German-layout QWERTZ keyboard). In the (online) Experiment 2, participants were instructed to use the numbered keys 1, 2, 3, 4 located on top of the keyboard. These keys were uniquely associated with the numbers 1 to 4 displayed on the monitor, so that the number 1 (or key F) corresponded to the little finger, 2 (or key T) corresponded to the ring finger, 3 (or key Z) corresponded to the middle finger, and 4 (or key J) corresponded to the index finger. In Experiments 1 and 2, the screen displayed a static string of five numbers (4–1-3–2-4), for a certain duration depending on the experiment design. The task was to produce that sequence using the corresponding fingers as fast and accurately and as many times as possible throughout the practice period, i.e., as long as the sequence stayed on the screen. The string of numbers was displayed in white on a black background, surrounded by the outline of a rectangle, whose color changed with every keypress, from white to gray, or back from gray to white, as feedback that the keypresses were being registered. In Experiments 3, 4, and 5, only the first number of the sequence was surrounded by a white square, and the display of the five numbers changed with every keypress, replacing the first number with the second, the second with the third, and so on, and adding a new number as the fifth. This was done to accommodate nonrepeating finger sequences, as described in detail in the corresponding section below. In the different experiments, the durations of the practice periods changed depending on the experimental manipulation, but the task instruction for practice periods remained the same throughout.

The practice periods were interleaved with rest periods of 10-s in some experiments. In Experiments 1, 2, 4, and 5, the screen displayed a static string consisting of five times the letter X (i.e., “XXXXX”) during rest periods, replacing the numbers. Participants were asked to fixate on the X’s while resting, and not move their fingers or any other body part. In the rest periods of Experiment 3, participants were shown the first sequence of the upcoming practice period, in red font (see details below).

#### Experiments 1 and 2 (with vs. without breaks, in-lab, and online study).

In order to test if offline periods during training resulted in additional learning, i.e., overall, more skill acquisition, Experiments 1 and 2 followed a between-subject design with two groups of participants: one group trained with interspersed breaks (With Breaks) whereas another group trained continuously for the same duration without interspersed breaks (No Breaks). We analyzed data from 85 in-lab participants (Experiment 1) and 358 online participants (Experiment 2; see Dataset Exclusion Criteria below for details on excluded datasets). During training, participants in the With Breaks group had to learn the sequence 4-1-3-2-4 via 10-s practice periods, interspersed with 10-s rest. Participants in the other group (No Breaks) had to learn the same sequence via continuous practice without breaks. The total practice duration was matched between groups. Following training, both groups took a break of 5 min, which allowed for washout of fatigue. Test sessions of 20-s each were introduced before the beginning of training, at end of training, and at the end of washout (Fig. 1*A*). The task during these test sessions was identical to the practice periods during which participants repeatedly performed the 4-1-3-2-4 sequence as frequently and accurately as possible. To ensure consistent test conditions across groups, each test session was preceded by a 3-s on-screen countdown, where participants were asked not to press any key. In order to explore potential group differences across longer training, a second block of training, washout, and tests was added. Experiment 2 (online) had the identical task design, with the exception that two attention checks were introduced during each of the 5-min washout periods, in order to ensure that participants did not move away from the keyboard. Each attention check required participants to press a key within the number of seconds shown on the screen. Participants in both experiments were motivated to perform to the best of their capacity by rewarding them bonus money based on the total number of correct sequences they performed throughout the experiment.

#### Experiments 3 and 4 (repeating vs. nonrepeating sequences, with and without advance information).

Experiments 3 and 4 tested whether the performance improvement across rest requires replay. To this end, one group each (“Nonrepeating”) in Experiments 3 and 4 performed sequences of finger movements which never repeated, neither within a given practice period, nor across the entire experiment. If MOGs are due to replay, they should vanish when sequences never repeat. This is because replay of any sequences that were previously encountered can only improve future performance of that same sequence, i.e., benefits mediated by this type of specific replay effects would require that sequences repeat across practice periods. To accommodate never-repeating sequences, we modified the practice periods to closely mimic a DSP (33) (discrete sequence production) task. Every practice period was 10-s in duration. During that time, participants always saw five numbers at a time on the screen (in white on a black background). The first number was surrounded by a square frame and cued the next required movement. Participants were instructed to press the key corresponding to that number as accurately and fast as possible. As soon as they pressed, the sequence of numbers moved to the left by one position, such that the number within the square disappeared, and the second number from the left moved into its position, while a new number appeared in the fifth position. Practice periods lasted 10 s, so that the participants paced the rate of cue presentation. The “Repeating” group of participants performed the same five-element sequence of numbers (4–1-3–2-4) repeatedly, whereas the Nonrepeating group of participants were presented with a set of five-element sequences that never repeated. Both groups completed 10 trials of alternating practice and 10-s rest periods. To mimic the 4-1-3-2-4 structure of the repeating sequence, the nonrepeating sequences were generated with some constraints: all four possible numbers (1-4) had to be present, with any one of these numbers presented twice in a sequence of five-elements, and consecutive repetitions of a single number or of a pair of numbers within the sequence were not allowed. These unique five-element sequences were organized into a long string such that the last number of a sequence was the same as the first number of the next sequence (e.g., “2-4-2-3-1-1-2-4-1-3...”), in order to match the Repeating group (“4-1-3-2-4-4-1-3-2-4...”). Participants of neither group were explicitly aware of the sequence demarcations.

In Experiment 3, both groups received advance information about the first sequence of an upcoming practice period, keeping it similar to the original studies (13–15), in which participants knew throughout rest periods which sequence they would have to perform next. Therefore, the 10-s rest periods displayed the first five cues of the upcoming practice period, in the form of a five-element sequence displayed in red. For the Repeating group, this sequence was always 4-1-3-2-4, and for the Nonrepeating group, it was the first sequence of the upcoming practice period.

In Experiment 4, on the other hand, the 10-s rest period preceding every practice period displayed “XXXXX” in red at the center of the black screen. This restricted participants from preplanning the first sequence during rest.

#### Experiment 5 (preplanning).

In this within-subject study, we tested if preventing planning of more than the next finger movement diminishes MOGs. All the sequences in all trials were nonrepeating. There were 40 trials, each having a 10-s rest period and a 10-s practice period, with two interleaved conditions: Window size 5 and Window size 1, which allowed to preplan up to five upcoming movements or only the single next movement, respectively. The two conditions switched every four trials. In the Window size 5 condition, the practice period showed a sequence of five numbers in white, with the leftmost number being surrounded by a white rectangle. Thus, participants were able to know the full sequence and preplan up to five finger movements before initiating the first finger movement at the start of each practice period, as well as plan online. Participants were instructed to respond as correctly and as quickly as they could, by pressing the key corresponding to the number within the rectangle, using their left hand’s corresponding finger. As soon as they pressed, the sequence shifted one number to the left, as described for Experiments 3 and 4. Participants were instructed to respond to the number within the rectangle at all times. In the Window size 1 condition, the practice period showed a single number within the white rectangle, followed by four X’s to its right (for example, “3 X X X X”), thus removing the possibility for planning any movement beyond the single next movement. Participants were instructed to respond as correctly and immediately as possible. As soon as they pressed a key, the white rectangle showed the next number to be pressed, while the X’s stayed in the same position. All presented numbers in both conditions belonged to five-element sequences, unknown to participants. All practice periods were timed to 10-s, so the number of sequences that each participant encountered depended on their speed of responses. Sequences were generated in a way that there was no single number, or pair of numbers, that appeared consecutively within the same sequence. We also ensured that all four fingers were represented in each sequence at least once, and that any one finger number appeared twice in the combination in order to obtain a five-element sequence. Each 10-s practice period was preceded by a 10-s rest period. During rest, participants saw “XXXXX” in white on a black background, and were asked to fixate on the “XXXXX” without making any movement, and rest their fingers on the keys. Thus, any preplanning in the Window size 5 condition could only occur once the practice period began and the five numbers were displayed.

##### Data analysis.

###### Dataset exclusion criteria.

Out of the complete datasets collected for each experiment, the following number of datasets were discarded from analysis for the respective experiments (see Exclusion Criteria table below): 4 from Experiment 1, 55 from Experiment 2, 8 from Experiments 3 & 4, and 8 from Experiment 5.

In Experiment 2, all participants’ data were screened to check for completion, attention tests and time of initiation after the 5-min washout break periods. Fully complete datasets which passed both attention tests for both washout periods, and showed initiation times less than 2.5 s from the start of the postwashout test, were included for analysis.

**Table:** 

Experiment	Reason for exclusion (No. of participants excluded)
Experiment 1 (Breaks vs. No Breaks): in-lab study	Anomalous performance–zero correct keypresses after baseline and 1st training (1) Anomalous performance–zero correct keypresses in the last test T5, although they performed well throughout (1) Problems during data acquisition–incorrect keyboard layout (English instead of German), failure to follow instructions (2)
Experiment 2 (Breaks vs. No Breaks): online study	Failed attention checks during 5-min breaks (5) Poor task commitment–took more than 2.5 s to make first response after task started post-5 min breaks (38) Unable to follow task instructions–pressed wrong keys in a pattern throughout the experiment (12)
Experiments 3 & 4 (Repeating vs. Nonrepeating sequences, with & without advance information)	Due to poor commitment to the task–zero correct sequence performed in multiple trials (6 & 2)
Experiment 5 (preplanning)	Due to technical issues during data acquisition leading to invalid datasets (7) Due to poor performance, pressing wrong keys on the keyboard (1)

All recorded data were stored anonymously and with the consent of participants under GDPR regulations. Information about identity and timings of the keypresses were recorded, for every practice and rest period in all the experiments. Most of the data analyses were done with the same methods as used by previous studies (13–15).

###### Number of correct keypresses.

While the task required producing as many fully correct sequences as possible during each practice period, errors likely resulted in sequences that were only partially correct (for example, four of the five required keypresses correct). Quantifying performance purely as the number of fully correct sequences would therefore disregard part of the task-appropriate performance, i.e., sequences that were only partially correct. To avoid this, we computed the number of correct keypresses per practice period, rather than the number of fully correct sequences (13). Our approach to calculate the number of correct keypresses differed between Experiments 1 and 2 on the one hand, and Experiments 3-5 on the other hand. In Experiments 1 and 2, the same sequence was required throughout the entire experiment, and we did not cue single keypresses, one at a time. As a result, when they made an error, participants were free to complete the current sequence despite the error, or restart. To accommodate this, we used a sliding window approach. We first calculated keypresses that were part of fully correct sequences in a given 20-s test period. For calculating the number of keypresses which were not part of a fully correct sequence, the positions of all keypresses in fully correct sequences were replaced by NaN (as a placeholder), and we then moved a five-element sliding window to match keypresses in the current window with the required sequence position-wise, according to the following criterion. If at least three keypresses in the current five-element window matched the required sequence by position, these keypresses were counted as correct keypresses, and they were replaced by NaN before the sliding window moved on. The reason why we set the threshold to at least three correct keypresses was to only count correct attempts to perform a fully correct sequence, and disregard instances when participants repeated, e.g., the first one or two elements of the sequence. In the case of Experiments 3, 4, and 5, in which keypresses were cued individually, one at a time, all correct responses to the currently valid cue were counted as correct keypresses.

In Experiments 1 and 2, the continuous training durations of the group with no breaks were binned into three 10-s bins, for comparison with the group with breaks. The number of correct keypresses was calculated in the same manner as for the 20-s test bins, as explained above. Thus, the sliding window used for detecting correct keypresses matched keys to the sequence based on their position. As a result, it could miss detecting the first keypresses of a bin, if the bin did not start with a correct “4-1-3-..” initiation, due to the artificial binning.

In the *SI Appendix*, we present an alternative binning method, referred to as the “credit system” (outlined in *SI Appendix*, Fig. S1 *E* and *K* and Table S2, subheading 3), where credits are allocated for extra keypresses at the end of a bin (for both groups), and at the beginning (only for the group with no breaks). This method offered an adjustment for the group with no breaks, whose sequences might have been interrupted by the artificial binning. The method yielded very similar results, as shown in *SI Appendix*, Table S2, subheading 3.

In Fig. 1 *C* and *E*, all 20-s test periods were divided into two 10-s bins, with the average number of correct keypresses across the two bins representing a single value for each 20-s period. This binning was also applied for statistical purposes, specifically to T1 and T2 when examining practice effects, where they serve as baselines for comparison with the binned training periods. This approach ensured consistency in keypress counting and accounted for any variations that could arise from the binning process. However, the primary statistical comparisons between groups at each of the five test periods are based on the total number of correct keypresses across the full 20-s test durations, without artificial binning.

###### Tapping speed of a sequence.

Tapping speed of a completely correct sequence was evaluated as the inverse of the mean of the four interpress intervals separating the five keypresses that constituted a fully correct sequence (keypresses per second).

###### Micro-online and micro-offline gains.

Using the same method as Bönstrup et al. (13, 14), the difference in tapping speed between the first and the last correct sequence within a practice period was evaluated as “micro-online gains,” and the difference in tapping speed between the last correct sequence of a practice period and the first correct sequence of the next practice period was evaluated as “micro-offline gains,” i.e., MOGs. The sum of these performance changes across all trials, results in the “online gain” or “offline gain” for each participant.

In Experiments 3, 4, and 5, we averaged the time between five consecutive responses that corresponded to a sequence (predefined by us as described above, but unknown to the participants), and obtained tapping speed for correct sequences by taking the inverse. The last five correct keypresses which were a part of a complete sequence were considered to be the last correct sequence of a practice period. For evaluation of the sum of offline performance improvements in both conditions of Experiment 5 (“Window size 5” or “Window size 1”), no MOGs were computed for the rest periods for which the condition changed (i.e., every fourth rest period was omitted). In Experiments 3 and 4, the sum of performance improvements across 10 trials was used, whereas for Experiment 5, the sum across 15 trials were used, after discarding the transition trials.

###### Movement initiation time.

This was evaluated as the time from the start of the practice period until the first response was made. Therefore, initiation times were evaluated only for the trials in which the first response was part of a fully correct sequence. The median across all trials of a condition, was used for analyses.

###### Statistical analysis.

For statistical testing of online and offline performance changes, i.e. MOGs, we computed one-sample *t* tests against zero in all experiments. In Experiments 1 and 2, we compared learning between groups via 2 × 5 repeated measures ANOVA (two groups, five test sessions). For the two training blocks, we computed two 2 × 4 repeated measures ANOVA (two groups, four time points: baseline test and three training bins), one for each training block. The baseline for each training block was the average of performance in the two 10-s bins which were a part of the test session that preceded the respective training block (i.e., first test session for the first training block, and third test session for the second training block). The Greenhouse–Geisser correction was used for all instances where the assumption of sphericity was violated. Interaction effects were followed up by *t* tests. We corrected all *P*-values in these cases for multiple comparisons using the Holm–Bonferroni method. For statistical comparison of MOGs between the Nonrepeating groups of Experiments 3 and 4, a Mann–Whitney *U* test was used to account for unequal variances between the groups of the two experiments, whereas an independent samples *t* test was used for comparison of groups within each experiment. In Experiment 5, we used Pearson’s correlation to correlate interindividual differences in tapping speed with differences in movement initiation times between conditions (“Window size 5” and “Window size 1”). All statistical analyses were conducted in JASP (Version 0.17.1.0, jasp-stats.org) and MATLAB (MathWorks).

## Supplementary Material

Appendix 01 (PDF)

## Data Availability

Human behavioral experimental data have been deposited in OSF (https://osf.io/5d6ew/?view_only=bae01453135847bbb3526b731271f86a) (50).
