# Supplementary material for: Micro-offline gains do not reflect offline learning during early motor skill acquisition in humans
Source: Proc Natl Acad Sci U S A. 2025 Oct 28;122(44):e2509233122. doi: 10.1073/pnas.2509233122 (PMC12595466; doi:10.1073/pnas.2509233122)
Supplement: Supplementary file 1 — Appendix 01 (PDF) [file pnas.2509233122.sapp.pdf]

SUPPORTING INFORMATION

for

"Micro-offline gains" do not reflect offline learning during early motor skill acquisition in humans

Anwesha Das[1,2,*], Alexandros Karagiorgis[1,2], Jörn Diedrichsen[3,4,8], Max-Philipp Stenner[1,2,5,6,8,*], Elena Azañón [1,2,5,6,7,8]

[1] Leibniz Institute for Neurobiology, Magdeburg, Germany

[2] Faculty of Medicine, Otto-von-Guericke University, Magdeburg, Germany

[3] Western Institute of Neuroscience, University of Western Ontario, Ontario, Canada

[4] Departments of Computer Science, Statistical and Actuarial Sciences, University of Western Ontario, Ontario, Canada

[5] Center for Behavioral Brain Sciences, Magdeburg, Germany

[6] Center for Intervention and Research on adaptive and maladaptive brain Circuits underlying mental health, Jena-Magdeburg-Halle, Germany

[7] Department of Psychology, Otto-von-Guericke University, Magdeburg, Germany

[8] Contributed equally as senior authors

* Corresponding authors: max-philipp.stenner@med.ovgu.de, anweshaz1997@gmail.com

This PDF file includes:

Supporting text
Figures S1 to S4
Tables S1 to S3
Reference S1

1. Training sessions										
bin	***In-lab study***					***Online study***				
	BF_{10}	*t(83)*	p_{unc}	p_{corr}	*d*	BF_{10}	*t(356)*	p_{unc}	p_{corr}	*d*
T1 (mean 2 bins)	.281	-0.71	.483	1	-0.15	.71	-1.94	.054	.216	-0.21
Training 1_bin1	.342	0.97	.335	1	0.21	.44	1.66	.098	.294	0.18
Training 1_bin2	.609	1.51	.135	.540	0.33	129.38	3.85	<.001	.008	0.41
Training 1_bin3	2.049	2.26	.026	.182	0.49	1109.23	4.41	<.001	.008	0.47
T3 (mean 2 bins)	.229	0.17	.867	1	0.04	.16	-0.83	.407	.620	-0.09
Training 2_bin1	2.848	2.43	.017	.136	0.53	.19	1.02	.310	.620	0.11
Training 2_bin2	1.413	2.06	.043	.258	0.45	1.85	2.40	.017	.085	0.25
Training 2_bin3	1.010	1.86	.067	.335	0.40	6.83	2.92	.004	.024	0.31
2. Test sessions										
bin	***In-lab study***					***Online study***				
	BF_{10}	*t(83)*	p_{unc}	p_{corr}	*d*	BF_{10}	*t(356)*	p_{unc}	p_{corr}	*d*
T1 (baseline)	.241	-0.37	.711	1	-0.08	.969	-2.10	.036	.180	-0.22
T2 (end 1st tr.)	.340	0.97	.338	1	0.21	.174	0.91	.364	1	0.10
T3 (1st retention)	.227	0.07	.944	1	0.02	.148	-0.71	.481	1	-0.08
T4 (end 2nd tr.)	.248	0.46	.650	1	0.10	.161	-0.81	.417	1	-0.09
T5 (2nd retention)	.227	0.10	.920	1	0.02	.120	-0.25	.804	.962	-0.03

Table S1. Experiments 1 (in-lab) & 2 (online), in reference to Figure 1 panels C and E: 1. Training sessions: Results from independent-samples t-tests comparing the number of correct keypresses between groups during training (bins 1-3, separately for the first and second training block) and pre-training baseline (T1 and T3). A 2x4 ANOVA (two groups and four time points: baseline and three training bins) revealed a main effect of time point for the first training block (in-lab study: F(2.4,199.8)=78.55, p<.001, $h^2_{partial}$=0.49; online study: F(2.9,1036.9) = 189.82, p<.001, $h^2_{partial}$= 0.35), as well as the second training block (in-lab study: F(2.8,229.7) = 24.84, p<.001, $h^2_{partial}$= 0.23; online study: F(2.9,1029.6) = 58.71, p<.001, $h^2_{partial}$= 0.15). Additionally, there was a group by time point interaction, indicating that this improvement differed between groups, both for the first training block (in-lab study: F(2.4,199.8) = 7.60, p<.001, $h^2_{partial}$= 0.08; online study: F(2.9,1036.9) = 27.19, p<.001, $h^2_{partial}$=0.07), and the second training block (in-lab study: F(2.8,229.7) = 6.59, p<.001, $h^2_{partial}$= 0.07; online study: F(2.9,1029.63) = 17.48, p<.001, $h^2_{partial}$= 0.05). **2. Test sessions:** Results from independent-samples t-tests comparing the number of correct keypresses between groups in each of the five test sessions (T1 - T5). We corrected p-values for multiple comparisons using the Bonferroni-Holm method (p_{corr}; p_{unc} represents uncorrected p-values). All tests reported in the table are two-sided.

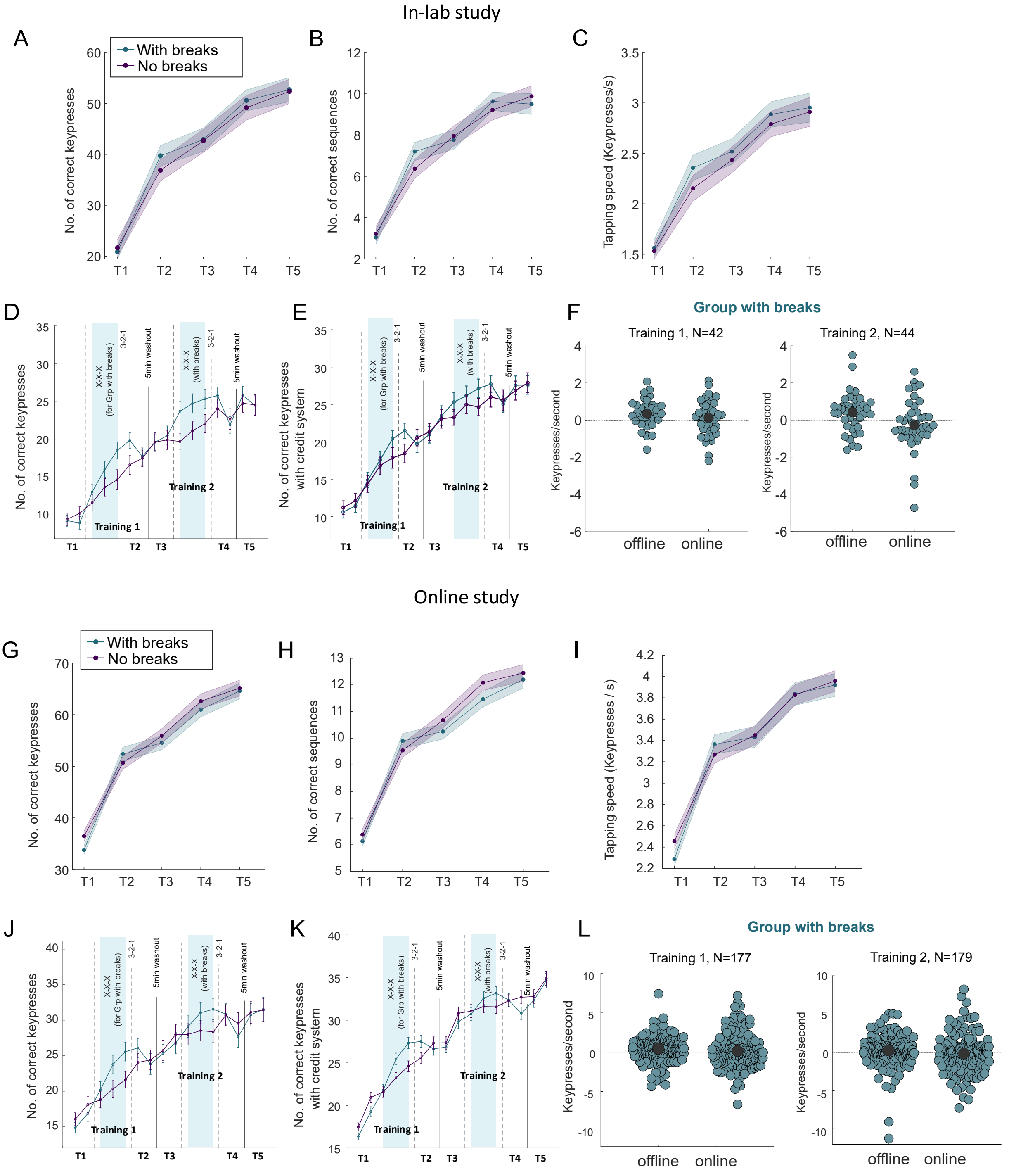

Figure S1, panels A-F. In reference to data from Experiment 1 (In-lab study, With breaks group, n = 44 & No breaks group, n = 41). Panels **A**, **B** and **C** display the number of correct keypresses, the number of correct sequences and the mean tapping speed of correct sequences, between the two groups across each 20-s test period. No significant differences were observed between the groups in any 20-s test period for these measures. Panels **D** and **E** present the number of correct keypresses for each 10-s bins (artificially binned) throughout the experiment, without (**D**) and with a credit system (**E**, see table S2 for an explanation of the credit system). Panel **F** shows the 'Micro-offline gains' for the group with breaks, during both training periods separately. Participants who did not exhibit gains across a single offline period in the respective trainings, could not be represented in the figure, therefore N=42 in the 1st training and N=44 in the 2nd training. **Figure S1, Panels G-L. In reference to data from Experiment 2 (Online study, With breaks group, n = 179 & No breaks group, n = 179).** Panels **G**, **H** and **I** display the number of correct keypresses, the number of correct sequences and the mean tapping speed of correct sequences, between the two groups across each 20-s test period. Panels **J** and **K** present the number of

correct keypresses for each 10-s bins (artificially binned) throughout the experiment, without (**J**) and with a credit system (**K**). Panel **L** shows the 'Micro-offline gains' for the group with breaks, during both training periods separately. Participants who did not exhibit gains across a single offline period in the respective trainings, could not be represented in the figure, therefore N=177 in the 1st training and N=179 in the 2nd training.

1. Results of ANOVA and independent samples t-test for the in-lab study (Figure S1, panels A–C)

Effect	***N correct keypresses***			***N correct sequences***			***Tapping speed***		
	F	*p*	$h^2_{partial}$	*F*	*p*	$h^2_{partial}$	*F*	*p*	$h^2_{partial}$
Test	250.08	<.001	0.75	166.60	<.001	0.67	170.49	<.001	0.69
Group	0.09	.761	< .01	0.04	.841	4.9e-4	0.34	.559	< .01
Test*Group	0.78	.504	< .01	1.52	.212	0.02	0.80	.481	0.01

Test #	***N correct keypresses***			***N correct sequences***			***Tapping speed***		
	t (83)	p_{unc}	*d*	*t (83)*	p_{unc}	*d*	*t (83)*	p_{unc}	*d*
T1	-0.37	.711	-0.08	-0.36	.721	-0.08	0.26	.791	0.06
T2	0.97	.338	0.21	1.32	.190	0.29	1.18	.240	0.26
T3	0.07	.944	0.02	-0.27	.792	-0.06	0.47	.639	0.10
T4	0.46	.650	0.10	0.64	.526	0.14	0.52	.606	0.11
T5	0.10	.920	0.02	-0.53	.597	-0.12	0.22	.829	0.05

2. Results of ANOVA and independent samples t-test for the online study (Figure S1, panels G–I)

Effect	***N correct keypresses***			***N correct sequences***			***Tapping speed***		
	F	*p*	$h^2_{partial}$	*F*	*p*	$h^2_{partial}$	*F*	*p*	$h^2_{partial}$
Test	591.54	<.001	0.62	466.55	<.001	0.57	424.42	<.001	0.55
Group	0.30	.584	8.43e-4	0.48	.488	<0.01	0.03	.864	8.3e-5
Test*Group	2.92	.030	0.01	2.70	.038	0.01	2.54	.056	0.01

Test #	***N correct keypresses***			***N correct sequences***			***Tapping speed***		
	t (356)	p_{unc}	*d*	*t (356)*	p_{unc}	*d*	*t (365)*	p_{unc}	*d*
T1	-2.10	.036	-0.22	-0.87	.388	-0.09	-1.86	.063	-0.20
T2	0.91	.364	0.10	0.92	.361	0.10	0.80	.425	0.09
T3	-0.71	.481	-0.08	-1.02	.307	-0.11	-0.12	.904	-0.01
T4	-0.81	.417	-0.09	-1.52	.131	-0.16	0.06	.955	0.01
T5	-0.25	.804	-0.03	-0.54	.590	-0.06	-0.25	.800	-0.03

3. Results of independent samples t-test for in-lab and online studies, using credit system (Figure S1 panels E & K)

**Time point**	***In-lab study***			***Online study***		
	t(83)	p_{unc}	*d*	*t(356)*	p_{unc}	*d*
T1 (mean of 2 bins)	-0.58	.564	-0.13	-2.09	.037	-0.22
Training 1_bin1	0.31	.755	0.07	0.29	.776	0.03
Training 2_bin2	0.43	.669	0.09	2.35	.019	0.25
Training 3_bin3	1.44	.153	0.31	2.97	.003	0.31
T3 (mean of 2 bins)	0.01	.990	0.01	-0.74	.457	-0.08
Training 2_bin1	1.24	.218	0.27	-0.31	.755	-0.03
Training 2_bin2	0.64	.523	0.14	0.93	.354	0.10
Training 2_bin3	1.46	.148	0.32	1.46	.146	0.15

4. Results of online and offline (MOG) performance improvements for each training separately, in the in-lab and online studies (Figure S1 panels F & L)								
Sums of improvements	***In-lab study***				***Online study***			
	t	p_{unc}	d	BF_{10}	*t*	p_{unc}	*d*	BF_{10}
Training 1 offline	3.06	.004	0.46	9.02	3.84	<.001	0.29	90.24
Training 2 offline	2.81	.007	0.42	5.06	1.81	.072	0.14	0.41
Training 1 online	0.79	.433	0.12	0.22	0.93	.353	0.07	0.13
Training 2 online	-1.41	.165	-0.21	0.41	-0.69	.493	-0.05	0.11

Table S2. The table presents the outcomes of repeated-measures ANOVAs and independent-samples t-tests for the in-lab study (**Figure S1, panels A–C**, Experiment 1) and the online study (**Figure S1, panels G–I**, Experiment 2). It shows effects of Test (5 20s tests), Group (with and without breaks), and their interaction for the number of correct keypresses, the number of correct sequences, and tapping speed. Below, independent-samples t-tests are reported for each test bin (T1–T5). Additional independent-samples t-tests are provided for individual training bins in both studies comparing the number of correct keypresses between the two groups during training, using a credit system to account for interrupted sequences during the artificial binning (**Figure S1, panels E and K**). When the keypresses were artificially split into 10-second bins, sequences might be divided between bins. For example, a correctly typed 5-element sequence might be split, with 3 keypresses falling in one bin and 2 in the next. According to our method for evaluating correct keypresses (see Data Analysis in the main manuscript), keypresses at the boundary of a bin would not be counted if fewer than three consecutive keypresses matched the sequence. This could also occur at the start of a bin if the sequence did not begin with "4-1-3...". To address such cases, we implemented an additional credit system. In this system, any instance of 2 or fewer correctly ordered keypresses at the end of a bin (for both groups) was added to the total correct keypress count for that bin. Additionally, at the start of the next bin, for the 'No Breaks' group only, we counted keypresses that corresponded to correct sequences interrupted by artificial binning. We observed similar results regardless of the method used. A 2x4 ANOVA (two groups and four time points: baseline and three training bins) revealed a main effect of time point for the first training block (in-lab study: $F(2.6,215.9) = 77.35$, $p<.001$, $h^2_{partial} = 0.48$; online study: $F(3,1068) = 182.3$, $p<.001$, $h^2_{partial} = 0.34$), as well as the second training block (in-lab study: $F(3,249) = 20.42$, $p<.001$, $h^2_{partial} = 0.20$; online study: $F(2.9,1027.4) = 54.30$, $p<.001$, $h^2_{partial} = 0.13$). Additionally, there was a group by time point interaction, indicating that this improvement differed between groups, both for the first training block (in-lab study: $F(2.6,215.9) = 2.94$, $p=0.041$, $h^2_{partial} = 0.03$; online study: $F(3,1068) = 15.30$, $p<.001$, $h^2_{partial} = 0.04$), and the second training block for the online study ($F(2.9,1027.4) = 5.94$, $p<.001$, $h^2_{partial} = 0.02$), and a trend towards significance for the in-lab study ($F(3,249) = 2.18$, $p=0.091$, $h^2_{partial} = 0.03$). Finally, results of online and offline performance improvements (i.e., micro- online and offline gains) are reported separately for the in-lab and online studies (Figure S1, panels F and L). One-sample t-tests against zero were conducted on the sum of online and offline performance improvements during each training (3 trials each, i.e., 3 online values and 2 offline values) separately, for the group with breaks; n=44 (df=43) & 179 (df=178) for the in-lab & online studies respectively. In Training 1, two participants in the in-lab study and two in the online study did not exhibit a single offline gain and were excluded, resulting in n=42 (df=41) and n=177 (df=176) for 'Training 1 offline' in the in-lab & online studies respectively. All tests reported in the table are two-sided (p_{unc} represents uncorrected p-values).

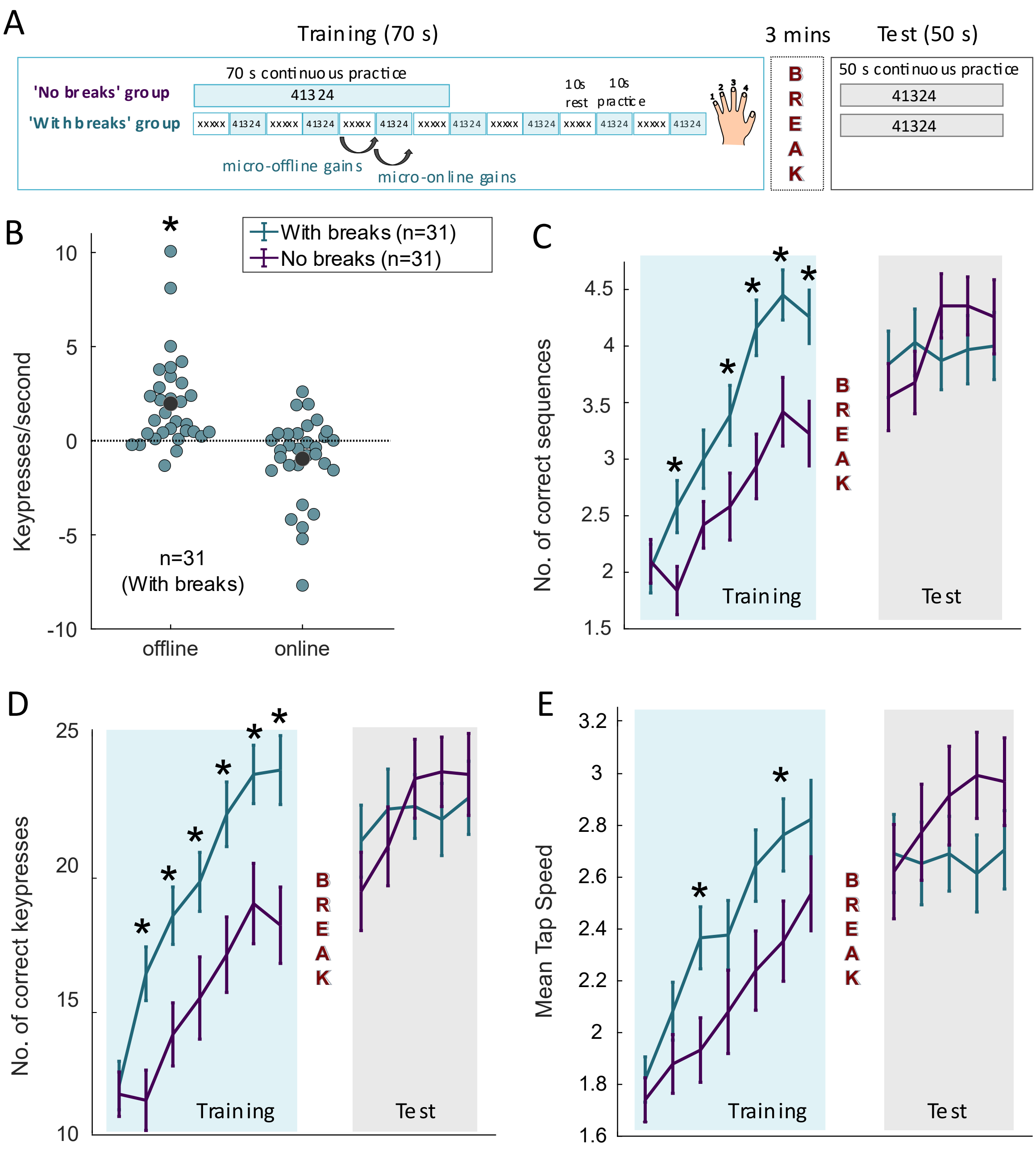

Figure S2. Additional Experiment S1. Training with breaks vs no breaks. Prior to running Experiments 1 and 2 reported in the main manuscript, we ran a similar study with n = 62 participants to test the impact of breaks during early motor skill acquisition (31 females, mean age 26.4 ± 3.5 years; With breaks, n = 31, No Breaks, n = 31). Panel **A** outlines the experiment design. In the group with breaks, participants practiced the sequence of finger movements 4-1-3-2-4 repeatedly through 7 trials, each lasting 10-s, followed by a 10-s rest. In contrast, the group without breaks practiced the sequence continuously for 70 seconds. Both groups then underwent a 3-minute washout period to eliminate fatigue or task-related effects. Skill retention was evaluated in a 50-s continuous practice block following the washout period. Participants were incentivized with bonus money based on the total number of correct sequences completed throughout the experiment, and they were instructed to be as fast but as accurately as possible. Analyses were performed in the same way as for the main experiments 1 and 2. We observed significant MOGs in the group with breaks. This group also showed superior performance in terms of correct keypresses during several 10-s intervals during training compared to the group without breaks (see statistics in Table S3). However, after the 3 minute washout, no group differences were observed during the 50-s test period. Panel **B** shows the micro-offline and micro-online gains during training for the group that trained

with breaks. There were significant micro-offline gains (MOGs) against zero (t(30)=4.453, p<.001, d=0.8, BF_{10}=239.153) and negative micro-online gains (t(30)=-2.371, p<.024, d=-0.426, BF_{10}=2.118) for the group that trained with breaks, across 7 trials. Panel **C** represents the number of correct sequences, panel **D** represents the number of correct keypresses and panel **E** represents the mean tapping speed of correct sequences, for both groups across training and test periods, in 10-s bins. All error bars represent the SEM.

**1. Training**									
Effect	***Number of correct keypresses***			***Number of correct sequences***			***Tapping speed per 10s***		
	F	*p*	$h^2_{partial}$	*F*	*p*	$h^2_{partial}$	*F (1,52)*	*p*	$h^2_{partial}$
Training	129.21	<.001	0.68	86.57	<.001	0.59	100.86	<.001	0.66
Group	4.76	.033	0.07	2.75	.103	0.04	1.17	.285	0.02
Training*Group	11.81	.001	0.16	9.25	.003	0.13	1.99	.165	0.04

**2. Test**									
Effect	***Number of correct keypresses***			***Number of correct sequences***			***Tapping speed per 10s***		
	F	*p*	$h^2_{partial}$	*F*	*p*	$h^2_{partial}$	*F (1,58)*	*p*	$h^2_{partial}$
Test	16.09	<.001	0.21	6.88	.011	0.10	6.38	.014	0.10
Group	0.07	.790	<.01	0.002	.968	2.8e-5	0.21	.650	0.004
Test*Group	3.40	.070	0.05	2.73	.104	0.04	4.58	.037	0.07

**3. Independent samples t-test comparisons**												
Time point	***Number of correct keypresses***				***Number of correct sequences***				***Tapping speed per 10s***			
	t(60)	p_{unc}	*d*	BF_{10}	*t (60)*	p_{unc}	*d*	BF_{10}	*t(60)*	p_{unc}	*d*	BF_{10}
Training bin 1	0.26	.793	0.07	0.27	-0.22	.824	-0.06	0.26	0.61	.547	0.16	0.32
Training bin 2	3.13	.003	0.80	13.60	2.36	.021	0.60	2.58	1.20	.234	0.33	0.50
Training bin 3	2.78	.007	0.71	6.09	1.76	.084	0.45	0.94	2.49	.016	0.64	3.29
Training bin 4	2.29	.025	0.58	2.27	2.03	.047	0.52	1.43	1.40	.168	0.36	0.59
Training bin 5	2.83	.006	0.72	6.70	3.25	.002	0.83	18.25	1.95	.055	0.50	1.27
Training bin 6	2.60	.012	0.66	4.13	2.75	.008	0.70	5.65	1.97	.054	0.50	1.29
Training bin 7	3.02	.004	0.77	10.33	2.65	.010	0.67	4.57	1.31	.195	0.34	0.54
Test bin 1	0.95	.348	0.24	0.38	0.70	.489	0.18	0.32	0.29	.772	0.08	0.27
Test bin 2	0.66	.510	0.17	0.31	0.88	.383	0.22	0.36	-0.49	.624	-0.13	0.29
Test bin 3	-0.55	.587	-0.14	0.29	-1.26	.212	-0.32	0.51	-0.94	.353	-0.24	0.38
Test bin 4	-0.95	.345	-0.24	0.38	-0.98	.333	-0.25	0.39	-1.70	.095	-0.43	0.86
Test bin 5	-0.43	.672	-0.11	0.28	-0.58	.562	-0.15	0.30	-1.16	.251	-0.29	0.46

Table S3. **Experiment S1 (Figure S2 panels C-E).** The table reports the results of a 2 x 2 repeated measures ANOVA (2 groups and 2 trials: 1st and 7th 10-s bins), in the number of correct keypresses, the number of correct sequences and the mean tapping speed, for the training trials, and below, for the test trials (2 groups and 2 trials: 1st and 5th 10-s bins). In addition, results of independent-samples *t*-tests are provided for group comparisons of correct keypresses, correct sequences and mean tapping speed between the two groups for all 10-s bins (artificially binned throughout the experiment). All tests reported in the table are two-sided (p_{unc} represents uncorrected p-values).

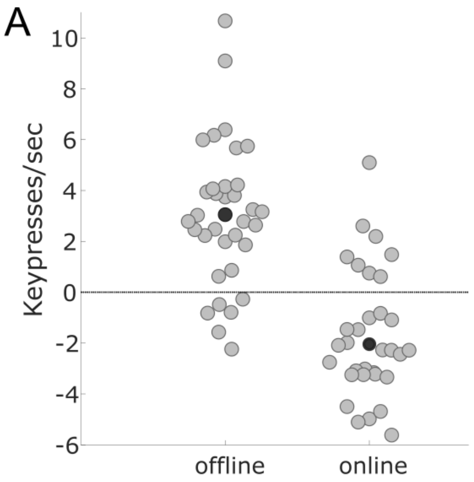

Figure S3: Experimental replication of Bönstrup et al.'s 2019 main paradigm. Data from a conceptual replication using the same task paradigm (N=34, 16 females, mean age = 27.3 ± 4.3 years) as in *Bönstrup et al. 2019, Current Biology*[1]*[S1]*. There were 36 trials with 10-s practice periods interleaved by 10-s rest periods. The sequence used was 4-1-3-2-4 and the feedback for every keypress was presented in the same way as *Bönstrup et al. 2019.* Participants heard white noise throughout the experiment, in order to prevent distractions as well as any learning as a function of keypress sound from the keyboard. The results show significantly positive 'micro-offline gains' (t(33) = 6.31, p = <.001, d = 1.08, BF_{10} = 41642.13) and significantly negative 'micro-online gains' (t(33) = -4.22, p = <.001, d = -0.72, BF_{10} = 150.37), across the first 12 and 11 trials[1], respectively.

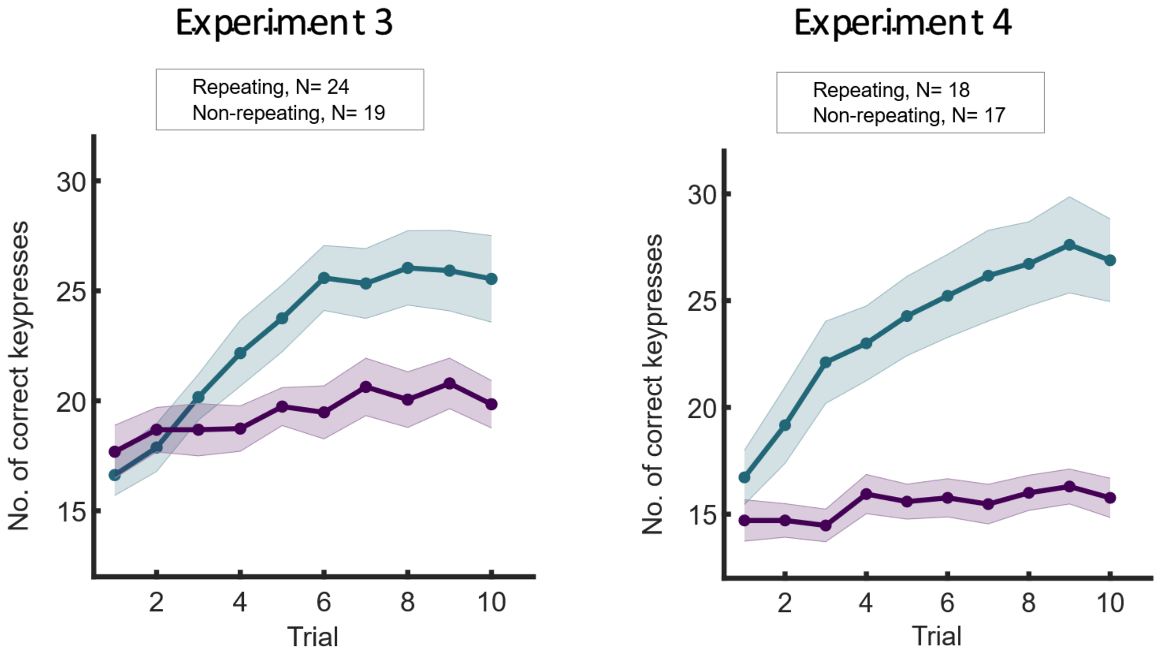

Figure S4: The number of correct keypresses performed in each trial, for experiments 3 and 4. The 'Repeating' group learnt to perform significantly more keypresses compared to the 'Non-Repeating' group across 10 trials, in both experiments. This was seen by a significant group x trial interaction, in both experiments: With advance information, F(3.582,146.861)=13.75, p<.001 $h^2_{partial}$=0.25; No advance information, F(4.7,155.3)=12.06, p<.001, $h^2_{partial}$=0.27. Error shading represent the SEM.

Accuracy metrics:

We computed the percentage of correct keypresses as a direct accuracy index and found no effect of group in this measure, neither in Experiment 1 (5 test sessions x 2 groups ANOVA, $F(1,83) = 0.31$, $p = .580$, $\eta^2_{partial} = 0.01$), Experiment 2 (5 test sessions x 2 groups ANOVA, $F(1,356) = 0.29$, $p = .590$, $\eta^2_{partial} < 0.01$), nor in Experiment S1 (independent samples t-test for 50s test session, $t(64) = 1.193$, $p=.237$, $d=0.30$, $BF_{10}=0.461$). Together with the absence of differences in tapping speed, number of correct keypresses, and correct sequences, these results confirm that participants did not shift their emphasis between speed and accuracy across conditions.

For Experiments 3 and 4, participants in the Repeating group produced more correct keypresses per 10 s than the Non-repeating group (independent samples t-test for mean number of correct keypresses across 10 trials, $t(41) = 1.98$, $p=.055$, $d=0.61$, $BF_{10}=1.39$ for Experiment, 3 and Welch t-test (as the equal variance assumption was violated in Student's t test), $t(24.007) = 4.53$, $p<.001$, $d=1.52$, $BF_{10}=221.78$, for Experiment 4. We did not find any differences in the percentage correct (number of correct key presses/number of pressed keys) across all 10 trials between Repeating and Non-repeating groups (10 trials x 2 groups Anova) in Experiment 3 (no effect of group, $F(1,41) = 2.66$, $p = .111$, $\eta^2_{partial} = 0.06$) and Experiment 4 (no effect of group, $F(1,33) = 1.08$, $p = .306$, $\eta^2_{partial} = 0.03$). Taken together, participants in the Repeating group produced more correct key presses in 10 seconds but made the same proportion of errors as the Non-repeating group.

Effect of monetary incentive across experiments:

We observed no performance difference between participants tested with or without additional monetary incentive (bonus payment based on performance). We compared the number of correct keypresses in the 7th trial of the 'With-breaks' group in Experiment S1 (where a bonus was provided) with the 7th trial of the 'Repeating' group in Experiment 4 (where no payment, neither bonus nor base compensation, was offered). Trial 7 was chosen because both experiments involved the same task and instructions up to trial 7, except for the bonus payment in Experiment S1 but not Experiment 4. Trial 7 thus allowed us to compare skill levels acquired with and without bonus payment under otherwise comparable conditions. Independent samples t-test for number of correct keypresses in the 7th trial between the 'With breaks' group of Experiment S1 (incentive given) and the Repeating group of Experiment 3 (no incentive given): $t(55) = -1.27$, $p =.21$, $d=-0.17$. This suggests that the observed absence of group differences in Experiments 1, 2, and S1 are not in any way related to the fact that participants received bonus payment based on their performance.

Percentage of correct keypresses:

Mean percentage of correct keypresses across all 11 sessions in Experiments 1 & 2: group with breaks = 92.96% & 94.32%, group with no breaks = 89.31% & 91.60%.

Mean percentage of correct keypresses across all 10 trials in Experiments 3 & 4: Repeating group= 97.73% & 97.99%, Non-repeating group = 96.35% & 97.11%.

Mean percentage of correct keypresses across 22 trials of each condition in Experiment 5: Window size 5 trials = 95.83% ; Window size 1 trials = 95.75%.

Supplemental References:

S1. Bönstrup, M., Iturrate, I., Thompson, R., Cruciani, G., Censor, N., and Cohen, L.G. (2019). A Rapid Form of Offline Consolidation in Skill Learning. Current Biology *29*, 1346-1351.e4. https://doi.org/10.1016/j.cub.2019.02.049.
